# Collaborative Care for patients with severe borderline and NOS personality disorders: A comparative multiple case study on processes and outcomes

**DOI:** 10.1186/1471-244X-11-102

**Published:** 2011-06-24

**Authors:** Barbara Stringer, Berno van Meijel, Bauke Koekkoek, Ad Kerkhof, Aartjan Beekman

**Affiliations:** 1 Department of Psychiatry and EMGO institute, VU University Medical Center/GGZ inGeest, Amsterdam, the Netherlands; 2 Research Group Mental Health Nursing, Inholland University for Applied Sciences, Amsterdam, the Netherlands; 3 Propersona, Centre for Education and Science, ProPersona, Wolfheze, the Netherlands; 4 Research Group Social Psychiatry & Mental Health Nursing, HAN University of Applied Science, Nijmegen, the Netherlands; 5 Department of Clinical Psychology and EMGO institute, VU University Medical Center, Amsterdam, the Netherlands

## Abstract

**Background:**

Structured psychotherapy is recommended as the preferred treatment of personality disorders. A substantial group of patients, however, has no access to these therapies or does not benefit. For those patients who have no (longer) access to psychotherapy a Collaborative Care Program (CCP) is developed. Collaborative Care originated in somatic health care to increase shared decision making and to enhance self management skills of chronic patients. Nurses have a prominent position in CCP's as they are responsible for optimal continuity and coordination of care. The aim of the CCP is to improve quality of life and self management skills, and reduce destructive behaviour and other manifestations of the personality disorder.

**Methods/design:**

Quantitative and qualitative data are combined in a comparative multiple case study. This makes it possible to test the feasibility of the CCP, and also provides insight into the preliminary outcomes of CCP. Two treatment conditions will be compared, one in which the CCP is provided, the other in which Care as Usual is offered. In both conditions 16 patients will be included. The perspectives of patients, their informal carers and nurses are integrated in this study. Data (questionnaires, documents, and interviews) will be collected among these three groups of participants. The process of treatment and care within both research conditions is described with qualitative research methods. Additional quantitative data provide insight in the preliminary results of the CCP compared to CAU. With a stepped analysis plan the 'black box' of the application of the program will be revealed in order to understand which characteristics and influencing factors are indicative for positive or negative outcomes.

**Discussion:**

The present study is, as to the best of our knowledge, the first to examine Collaborative Care for patients with severe personality disorders receiving outpatient mental health care. With the chosen design we want to examine how and which elements of the CC Program could contribute to a better quality of life for the patients.

**Trial registration:**

Netherlands Trial Register (NTR): NTR2763

## Background

A personality disorder is a severe and complex psychiatric illness. The borderline personality disorder (BPD) and the personality disorder not otherwise specified (NOS) both belong to the two most prevalent personality disorders. The lifetime prevalence of borderline personality disorders is estimated at 1-2% in the general population, whereas in patient samples the prevalence is approximate 10-20%. The personality disorder NOS has an estimated prevalence of 8-13% in patient samples [1,2].

Structured psychotherapy is recommended as the preferred treatment of personality disorders. Several studies report modest positive treatment results [3-9]. Psychotherapy contributes to higher quality of life, reduced psychopathology and destructive behaviour, and sustainable changes in personality.

A substantial group of patients, however, does not benefit from these psychotherapies [10-13]. Besides limitations in availability of these therapies, some patients do not meet the inclusion criteria or they drop out during treatment. Others need more psychosocial support for their many complex social problems. Most patients who do not benefit have a chronic and unstable course of illness with disruptive and destructive behaviour [10,13,14]. They put a high demand on the health care services provided for rather long, but often discontinuous periods of time [15]. These patients often receive community mental health care (often referred to as a team: CMHC team), mostly provided by (community) mental health nurses [10,14]. The treatment delivered by CMCH teams is, however, not standardized and highly unstructured [16,17].

Research indicates that especially nurses in particular experience caring for people with severe (borderline) personality disorders as highly stressful [18-21]. Strong emotional responses towards the patient arise frequently, particularly when the disruptive behaviour of the patient is unpredictable and difficult to understand. This contributes to condemnation of the patient by the nurse and a less empathic attitude. Ambivalent care seeking of these patients, shifting between dependency from and condemnation of professionals, can be explained out of their disorder and the irregular course of the therapeutic process. This same ambivalent care seeking, however, is difficult for care providers to accept and to cope with and it often leads to ineffective professional behaviour [22,23]. On the one hand, while balancing between autonomy and safety of the patient, nurses easily feel forced and responsible to protect the patient. Nurses may apply restrictive interventions to control the patient's destructive behaviour [24-26]. On the other hand, nurses may underestimate the needs and disabilities of their patients and perceive them as able but unwilling to change [27,28]. To keep the balance between playing a waiting game on the one hand, and being overly supportive and protective on the other hand is considered to be difficult with regard to these patients [13,27]. Studies reveal that patients and care providers set different priorities during treatment, including the specific needs of patients that require attention [29-33]. These, at times, conflicting priorities can cause miscommunication between patients and care providers and, hence, adversely affect outcomes of care [29,31].

As a response to these challenges, we developed a structured easily accessible intervention program for this subpopulation of patients, provided by (community) mental health nurses. For this intervention program we have adapted the principles of Collaborative Care (CC) [34-36]. Collaborative Care Programs originated in somatic health care to increase shared decision making and to enhance self management skills of chronic patients. Collaborative relationships come into existence when patients, their informal carers, and care providers have shared goals and mutual understanding of roles, expectations and responsibilities. As a consequence of more effective self management, patients report that their quality of life improves, because they feel they can better cope with problems derived from their disorder [35,36]. To date, Collaborative Care Programs (CCP) have proven to be effective for depressive and bipolar disorders [37-45].

Nurses have a prominent position in Collaborative Care Programs as they function as collaborative care managers. In this position they are responsible for optimal continuity and coordination of care. To optimize the continuity and coordination of care, intensive partnership working is needed within a *Collaborative Care team *(CCT). The CCT consists of the patient, his/her informal carer, the nurse, and the psychiatrist and/or psychologist. The CCT can optionally be expanded with others who possibly could contribute to effective treatment and care of the patient. The CCT lends support to the patient and it is in this team that crucial decisions regarding treatment will be made.

A Collaborative Care Program for patients with severe personality disorder has as to the best of our knowledge not yet been developed or tested. In this stage of intervention development, insights in both the feasibility and as well as the preliminary results of the intervention are needed. Therefore, we combine quantitative and qualitative data in a comparative multiple case study, which makes it possible to test the feasibility of the CCP in clinical practice, and also provides insight into the preliminary outcomes of CCP [46,47]. This study functions as a pre stage for a future RCT. The following research objectives are formulated:

(1). To describe the processes of the application of a Collaborative Care Program for patients with a severe borderline or NOS personality disorder in comparison with Care as Usual (CAU) from the perspective of patients, their informal carers and nurses;

(2). To describe the preliminary outcomes of the CCP in comparison with Care as Usual;

(3). To explain which characteristics of the CCP are indicative for the occurrence of positive or negative outcomes compared to CAU.

## Methods/Design

### Design

A comparative multiple case study may be used for the thorough evaluation of complex intervention programs [46-48]. The research generates descriptive and explanatory data regarding treatment processes and outcomes of the intervention program. Different perspectives are integrated in the evaluation: the perspective of patients, their informal carers and nurses. In our multiple case study two treatment conditions will be compared: one functions as the experimental condition in which the Collaborative Care Program is provided; the other condition functions as the control condition in which Care as Usual (CAU) is offered. Different types of data collection will be used: questionnaires, documents, and interviews. A case is defined as the treatment process of one patient in which integrated data from the three perspectives (patient, informal carers and nurse) concerning the application and the outcomes of the CCP or CAU will be gathered and analysed.

Within a comparative multiple case study, data are analyzed at the individual case level, group level, as well as between groups level [46-48]. The process of treatment and care within both research conditions is described with qualitative research methods. Additional quantitative data provide insight in the preliminary results of the CCP compared to CAU. By means of data triangulation, the connection between the application and the preliminary outcomes of the Collaborative Care Program will be explained in comparison with Care as Usual. With a stepped analysis plan the 'black box' of the application of the intervention program will be revealed in order to understand which characteristics and influencing factors are indicative for positive or negative outcomes.

### Participants

#### Patients

Participants are recruited from two comparable community mental health care (CMHC) teams of a large mental health organisation in the Netherlands. One team is indicated as the experimental condition and the other as the control condition.

Both CMHC teams provide long-term outpatient care for patients with various severe mental disorders. Patients that will be included should be between 18 and 65 years of age, have a main diagnosis of borderline or NOS personality disorder (DSM-IV-TR), have a score of 15 or higher on the Borderline Personality Disorder Severity Index (BPDSI, range 0-90) [5,49] and have received psychiatric care for at least two years. Participants are required to speak and read Dutch sufficiently well to fill in the questionnaires.

Participants are excluded when they currently participate in a structured psychotherapeutic program or when it is expected they will participate in such a program within a period of nine months from the start of the study. All participants will be asked to sign for informed consent based on oral and written information about the research project.

#### Informal carers

The participating patients will be asked to give their permission for approaching one of their informal carers to also participate in the study. The carers need to be in contact with the patient (physically or by telephone/email) for at least one hour a week. When the collaboration with an informal caregiver impedes the treatment process or negatively influences the safety of the patient, carers can be excluded. This will only take place after consultation with the patient.

#### Nurses

Ten mental health nurses from the experimental condition and five nurses from the control condition will be included in the study. Participation takes place on a voluntary basis. Nurses who participate in the experimental condition will receive a three-days training in providing the Collaborative Care Program. Nurses in the control condition will offer Care as Usual.

### Selection of patients

The required number of cases for a multiple case study depends upon the heterogeneity among the cases (more heterogeneity requires more cases) and is therefore arbitrary. To take into account the variety in presentation of the disorder and the variety of problems, this study will include at least sixteen patients in each condition. This adds up to 32 cases.

### Intervention

#### Collaborative Care Program

This Collaborative Care Program is developed to improve the quality of care for patients with severe personality disorders within a community mental health care setting. The expectation is that the Collaborative Care Program (1) improves quality of life, (2) reduces destructive behaviour (suicidal, self harm, aggressive or addictive behaviour) and other manifestations of the (borderline or NOS) personality disorder, (3) improves mastery of the patient, and (4) enhances satisfaction with care by both patients and informal caregivers. Finally, we aim for a positive effect on attitudes, knowledge and skills of nurses.

**Figure 1:**
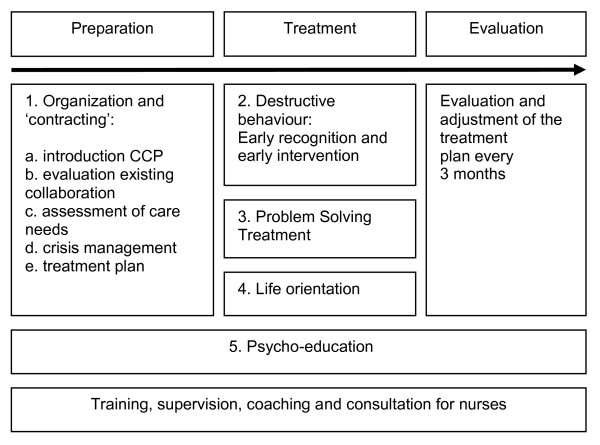
**the five integrated components of the Collaborative Care program**.

Collaborative Care for patients with severe borderline or NOS personality disorders consists of five integrated components (see Figure 1). The different components of the execution stage can be applied in a flexible order, dependent on the priorities in unmet needs and the preferences of the patient. Although CCP offers a goal-oriented structure, it comes to the professionalism of the nurses to adjust this structure to the preferences of the patient, the patient's cognitive capacities, and to the extent of illness insight/acceptance of each individual patient. The different components of the CCP will be briefly elucidated. The Collaborative Care Program is elaborated in a workbook for patients and nurses and in a separate manual for nurses. More detailed information about the content of the CC Program is available (see Additional file 1).

##### 1. Organization and contracting

A Collaborative Care Team will be put together for adequate coordination of care, with optimal collaboration between the main team partners: i.e. the patient, his/her informal carer(s), a psychiatric nurse and a psychiatrist and/or psychologist. Because discontinuity of care increases the risk of drop-out and a negative course of the psychiatric illness (with possible suicide as the extreme negative outcome), Collaborative Care demands pro-active collaboration between all partners to minimize this discontinuity of care. The nurse is responsible to inform and involve all those partners, whenever necessary. The execution stage of the CCP should not start before a treatment plan is established to which all collaborative partners commit [37,40,42]. Due to the ambivalent care seeking of most of the patients, this stage is therefore crucial and requires a careful preparation. This preparation stage consists of several activities (see Additional File 1).

Within Collaborative Care the patient is perceived as the one who shapes his own life, and hence his own treatment. Active involvement of the patient is required to reach the objectives of improved self management skills and shared decision making [36]. Patients, however, often have a long history of contacts with health care providers, with divergent success. To learn from previous experiences, an inventory is made of life events and of former treatments, based on the medical record. This inventory will be discussed with the patient and with the other members of the Collaborative Care Team to identify effective coping strategies with life events, effective elements in treatment, and relationships. Patients are invited to express their expectations about care providers and treatment and to speak aloud about disappointing (sometimes even traumatic) experiences, which still may hamper the relationships with care providers. Informal carers are invited to share their view upon past life events and expectations with regard to collaboration and treatment. Mutual expectations and responsibilities are made explicit between patients, informal carers and care providers, in order to promote a strong relationship [26,50]. The agreements about the collaboration are recorded in the treatment plan.

Health care needs will be assessed with the Camberwell Assessment of Needs (CAN) [51]. Based on the CAN results priorities in treatment goals will be set within the Collaborative Care Team. Unmet needs, goals and related activities are recorded in the treatment plan. In anticipation of possible crises, a crisis card will be compiled [26]. The use of a crisis card fits in the philosophy of collaborative care because it communicates that patients are (at least partly) able to cope with crisis themselves. If not, a back up of professional care is always available 24/7. The content of the crisis card is communicated with the crisis services and informal carers. Additionally, contracting will be used, based on shared decision making about the collaboration, the treatment process and the treatment plan [26,50]. The final step in the preparation stage is to confirm the goals, activities and agreements about collaboration in the treatment plans, which are evaluated every three months.

##### 2. Destructive behaviour

To reduce destructive behaviours a method of early recognition and early intervention will be implemented [26,52-54]. These destructive behaviours may have different forms: suicidal, self harm, addictive or aggressive behaviours. The central aim of the intervention strategy is the recognition of early warning signs (thoughts, feelings and/or behaviours) of the destructive behaviour of the patient. The aim is to help the patient gain a better insight in the process of destructive behaviour and to enhance coping with this behaviour. A relapse prevention plan will be drafted in which early signs are described, as well as actions how to respond to raising stress, despair and imminent crisis.

##### 3. Problem Solving Treatment

To reduce daily life problems Problem Solving Treatment (PST) will be applied [55]. The amount of daily life problems is often overwhelming in this subpopulation of patients, through which they may loose their sense of control. Learning and applying problem solving skills regarding daily problems enhances mastery and may result in a better quality of life. Mastery reflects the extent to which individuals perceive themselves in control of forces that significantly impact on their lives. PST has proven to be effective in different studies and is part of different treatments for personality disorders [50,55,56]. It is an essential element of Collaborative Care Programs [34,39,45].

##### 4. Life orientation

As counterbalance to the prominent attention to problems and destructive behaviour, the focus of CCP is also aimed at a more positive orientation in a person's life. Elements of Solution Focused Treatment will therefore be used to encounter and expand positive experiences which is expected to be stimulating for a renewed and more positive life orientation [57,58].

##### 5. Psycho-education

By means of psycho education, the patient (and their carer) is provided with knowledge about his or her psychological condition, the causes and consequences of that condition, ways of coping with it, and the treatment possibilities including the expected effects of it. Patients and their carers also will be prepared to the enduring character of the illness and to expected relapses. Psycho education is an integral element of Collaborative Care [36,39,42].

### Treatment integrity

The nurses who participate in the experimental condition will receive a three-day training program from three of the authors (BS, BvM en BK) in the principles and skills of the CC Program. During the provision of the CC Program, supervision for the nurses will be provided for continuing education on attitude and skills. Bi-weekly individual consultation and coaching (by telephone or email) will be offered based on the work sheets of the workbook and the manual to further support treatment integrity. Supervision, consultation and coaching are provided by the first author (BS).

### Control Condition

Patients in the control condition receive care as usual from their current care providers. During the study period, nurses in both conditions are not permitted to receive any extra training that might interfere with the content of the CCP.

#### Data collection

There are three measurements in this study: when participants enter the study (T0), after five months (T1) and after nine months (T2). To achieve the formulated objectives of this study, the data collection is divided into two parts. Quantitative data are collected with questionnaires to describe the outcomes of the CC Program in comparison with the CAU (summarized in table 1). Qualitative data, such as interviews, and records of the supervision sessions are used to analyse the implementation process of the application of the CCP in comparison with CAU. Data will be collected among patients, their informal carers and nurses, as mentioned below.

**Table 1:** Summary of the used questionnaires.

	Questionnaires		
Outcome indicator	*Patients*	*Informal caregivers*	*Nurses*
Quality of Life	▪ Manchester Short Appraisal (MANSA)		
Psychopathology	▪ Borderline Personality Disorder Severity Index (BPDSI) ▪ Structured Clinical Interview for DSM-IV Personality Disorders (SCID-II)		
Destructive behaviours	▪ BPDSI ▪ Beck Scale for Suicide Ideation (BSSI) ▪ CAGE questions-adapted to include drugs (CAGE-AID)		▪ Suicide Behaviour Attitude Questionnaire (SBAQ) ▪ Attitudes Towards Deliberate Self-Harm Questionnaire (ADSHQ)
Health care use	▪ Trimbos/iMTA questionnaire for Costs associated with Psychiatric Illness (Tic-P)		▪ process forms
Psychosocial symptoms	▪ Brief Symptom Inventory (BSI)		
Satisfaction	▪ Consumer Quality-index (CQ-index)	▪ CQ-index	
Therapeutic Alliance	▪ *S*cale *t*o *A*sses Therapeutic *R*elationships in Community Mental Health Care (STAR)		▪ STAR
Mastery	▪ Pearlin Mastery Scale (PMS)		
Involvement/social support		▪ Involvement Evaluation Questionnaire (IEQ)	

### Questionnaires for patients

#### Sample characteristics

Information will be gathered at baseline on demographic characteristics (age, gender, education level, marital status, work and ethnicity), history of illness, current medication use and diagnostic characteristics (DSM-IV Axis II by means of the Structured Clinical Interview for DSM-IV Personality Disorders (SCID-II) [59], the other axes are obtained from the medical records).

#### Primary outcome indicators

##### Quality of life

The Manchester Short Appraisal (MANSA) is a self-report scale, which measures quality of life. It is a short version (16 items) of the Lancashire Quality of Life Profile (LQoLP). Priebe et al. [60] found an adequate correlation between the results on both QoL scales.

##### Borderline Personality Disorder Severity Index (BPDSI)

The BPDSI is a DSM-IV BPD criteria-based semi-structured interview consisting of 70 items. It represents the current severity and frequency of the DSM-IV BPD manifestations. This instrument showed excellent psychometric features [5,49,61].

#### Secondary outcome and process indicators

##### Destructive behaviours

Four frequently observed destructive behaviours are measured. The BPDSI contains subscales measuring parasuicidal behaviour, including self harm, and aggressive behaviour. Additionally, the Beck Scale for Suicidal Ideation is used to measure suicidal thoughts, ideas and behaviours. It is a self-report scale of 21 items and has good psychometric properties [62,63]. The CAGE questions Adapted to Include Drugs (CAGE-AID) is a composed questionnaire describing the consequences of alcohol and drugs use [64].

##### Health care use

The Trimbos/iMTA questionnaire for Costs associated with Psychiatric Illness (TiC-P) is developed to measure health care consumption (part 1) and costs (part 2) [65]. In this study only part 1 of the questionnaire, concerning health care consumption, is used.

##### Psychosocial symptoms

The Brief Symptom Inventory (BSI) is a shorted version of the SCL-90 with 53 items (self report). Reliability and validity are almost identical to the SCL-90 [66].

##### Patient satisfaction

For the measurement of patient satisfaction the Consumer Quality-Index (CQ-Index) for outpatient mental health care is used [67]. It comprises items about information provision, involvement in treatment decisions, expertise and availability of professionals, and outcomes of treatment.

##### Quality of the therapeutic relationship

The *S*cale *t*o *A*sses Therapeutic *R*elationships in Community Mental Health Care (STAR) is a questionnaire which measures the quality of the therapeutic relationship [68]. A professional and patient version of the scale is available and a Dutch translation of this questionnaire will be used in this study.

##### Mastery

Pearlin and Schooler's Personal Mastery Scale (PMS, 1978) is a commonly used instrument to measure the external locus of control, also referred to as mastery. It consists of five items on a four point Likert scale (self report). The PMS has adequate validity and reliability [69,70].

### Questionnaires for informal carers

#### Process indicators

##### Carer satisfaction with care

For the measurement of carer satisfaction an adapted version of the CQ-index is used [67].

##### Involvement/social support

The Involvement Evaluation Questionnaire (IEQ) [71,72] is a self report list of 81 items, divided among seven sections. It measures consequences of care giving in informal carers.

### Questionnaires for nurses

#### Sample characteristics

Information is gathered at baseline on demographic characteristics (age, gender, education), working experience in mental health care and with this specific patient population.

#### Process indicators

##### Quality of the therapeutic relationship

Complementary to the patient's view on the quality of the therapeutic relationship, nurses will be asked to fill in the professional version of the STAR [68].

##### Attitudes towards destructive behaviours

The Suicide Behavior Attitude Questionnaire (SBAQ) consists of 21 items to be scored on visual analogue scales. Three subscale are differentiated: (1) feelings in relation with the care for suicidal patients, (2) professional skills and (3) the right for suicide [73].

Attitudes towards self harm are measured with the Attitudes Towards Deliberate Self-Harm Questionnaire (ADSHQ) as developed by McAllister *et al*. [74].

#### Process forms

Nurses in both conditions fill in process forms in which the number and content of contacts will be registered. In the experimental group items are added which provide additional insight in the treatment integrity. The process form follows the elements of the intervention and will systematically remind them on the structure and objectives of the CC Program.

### Qualitative data

#### Interviews

Individual interviews with patients, their carers and nurses (in this fixed order) will take place after the follow up measurement (T2). In the in-depth interviews the process of the application of the CC Program, and the relationship between this application and outcomes will be examined and compared to the application of CAU. In the interviews participants are first asked to reflect on the quantitative outcomes and on which changes they perceive as most beneficial. Subsequently, the underlying (neutrally formulated) principles of the CCP will be discussed, e.g. problem solving, coping with destructive behaviour, quality of the therapeutic relationship, and self-management. Next, exemplifications will be asked to identify characteristics of these principles which may explain the individual outcomes. Finally, the participants are asked to identify hampering or fostering components in the application of CCP or CAU.

The interviews will be audio taped and transcribed verbatim. The data will be analysed using WINMAX qualitative text analysis software. The credibility and dependability of the data will be ensured by peer debriefing, member checking, and thick descriptions [75].

#### Supervision records

During the execution of the CCP nurses receive supervision. It focuses on the individual application of the CCP and on the promoting and impeding factors regarding the execution of CC. The supervisions will be audio taped and transcribed verbatim. The records of these supervisions will be examined using content analysis.

#### Data analysis

A distinctive feature of a comparative multiple case study is the analysis of data on three different levels:

Firstly at individual case level, secondly at group level and thirdly at the level of the comparison between the two conditions. At case level the combined quantitative and qualitative data will be used to gain insight in how the application of the CCP in an individual participant has evolved and how this is related to the outcomes. Hence, in first instance a *within case analysis *of the data from different data sources and different perspectives will be made for each individual case. Secondly, within the experimental and the control condition *cross case analyses *will be performed to formulate statements about the observed processes and outcomes per condition. Cases will be subdivided in three categories: (1) a group of cases with positive outcomes; (2) a group of cases with none or minimal changes in outcomes and (3) a group of cases with negative outcomes. Within these three subgroups patient characteristics and the process of application will be compared to explain the different outcomes.

Finally, at an aggregated group level the observed differences in outcomes and process indicators will be examined between the experimental and the control condition (*cross case synthesis*) in order to assess the value of the intervention compared to care as usual and to explain differences in outcomes between the two treatment conditions.

### Qualitative analyses

To describe and understand the process of the application of the CCP versus CAU, the qualitative interviews with patients, their carers and nurses will be analyzed, following the three steps as described above. Beforehand, as preparation for the interviews, the supervision records will be analysed and the quantitative outcomes will be assessed at an individual level.

For the *within case analyses*, the data from the interviews are coded and categorized following the structure as described above. As said, for the *cross case analysis *the participants of both research conditions are divided in three subgroups. Based on the interview data, similarities and differences in the process of the application are described for the three subgroups. The different perspectives of patients, informal caregivers and nurses will be taken into account in this analysis. The degree, to which these perspectives differ from each other, might be indicative for the obtained outcomes. For the *cross case synthesis*, the data from the interviews will be examined to identify group differences between the two research conditions: Which statements do participants make about the underlying principles of the CCP? How do they value these principles? How do they value the outcomes of the CCP resp. CAU?

A content analysis of the supervision records will be performed to identify hampering and fostering characteristics in the process of the application of the CCP from a nursing perspective. For the *within case analysis *this information will be used as a preparation for the interviews. When performing the *cross case analysis and synthesis*, this information exemplifies and partially explains observed outcomes of the application of CCP.

### Quantitative analyses

The used questionnaires provide quantitative data about the outcome indicators from different perspectives. For the *within case analyses *the quantitative data are assessed to describe the individual outcomes. To facilitate the *cross case analysis*, differences in characteristics of the participants within the three subgroups are described. Descriptive analysis of the process forms will give additional information, which will be used for the *cross case analysis and synthesis*.

Statistical analyses will be performed to examine the differences at group level between the experimental and the control condition at the different measurements (*cross case synthesis*). Parametric and non-parametric comparisons of mean scores will be used. These analyses are used to identify preliminary results and to support the qualitative data. These quantitative data combined with the qualitative data provide insights in the value of CC and in the feasibility of the intervention from different perspectives.

## Discussion

A substantial group of patients with borderline or NOS personality disorders does, for different reasons, not participate in evidence based psychotherapeutic programs aimed at structural changes in personality and recovery. Poor quality of life, severe suffering, high risk of suicidal behaviour, and high health care use (and corresponding costs) of this population without access to these psychotherapies, justify the development of a structured, easy-accessible intervention program. Our Collaborative Care Program may function as a valuable alternative for the relatively unstructured treatment which dominates the care as usual within existent community mental health care teams [16,17]. Within these CMHC teams nurses are the main care providers, although they are not always equipped to meet this responsibility. Collaborative Care (CC) will offer them a structured method in providing care for patients with severe personality disorder.

The present study is, as to the best of our knowledge, the first to examine Collaborative Care for patients with severe personality disorders receiving outpatient mental health care. Currently, health care research on the outcomes of interventions is dominated by randomized clinical trials. However, depending on the development stage of interventions other designs are desirable and available [76,77]. With the chosen design we want to examine how and which elements of the CC Program could contribute to a better quality of life for the patients and whether it will give better results for their carers and the staff than care as usual. Based on the results of our study, the CC Program can be adapted in such a way that the chance for effectiveness will be maximized in a following RCT. This comparative multiple case study, hence, precedes the question of effectiveness. The start of this study is anticipated for January 2011 with results available in April 2012.

### Ethical considerations

This research project has been approved by the Medical Ethics Committee of the VU Medical Centre in Amsterdam, the Netherlands.

## Competing interests

The authors declare that they have no competing interests.

## Authors' contributions

BS is responsible for the initial draft of this article, and for the development, organization and implementation of the study. BvM and BK have contributed to the design and the development of the CC Program. The supervisors AB, AK, BK and BvM have reviewed the design and the workbook and manual of the CC Program, and revised earlier versions of the manuscript. All authors read and approved the final manuscript.

## Pre-publication history

The pre-publication history for this paper can be accessed here:

http://www.biomedcentral.com/1471-244X/11/102/prepub

## Supplementary Material

Additional file 1 **A Collaborative Care Program for patients with severe borderline or NOS personality disorders**. This additional file elucidates the content of the Collaborative Care Program in more detail. Click here for file
