# Supplementary material for: Collaborative Care for patients with severe borderline and NOS personality disorders: A comparative multiple case study on processes and outcomes
Source: BMC Psychiatry. 2011 Jun 24;11:102. doi: 10.1186/1471-244X-11-102 (PMC3135521; doi:10.1186/1471-244X-11-102)
Supplement: Additional file 1 — A Collaborative Care Program for patients with severe borderline or NOS personality disorders. This additional file elucidates the content of the Collaborative Care Program in more detail. [file 1471-244X-11-102-S1.DOC]

**A Collaborative Care Program for patients with severe borderline or NOS personality disorders.**

(Additional file to **Collaborative Care for patients with severe borderline or NOS personality disorders: a comparative multiple case study on process and outcomes)**

Barbara Stringer, Berno van Meijel, Bauke Koekkoek, Ad Kerkhof, Aartjan Beekman

**Introduction**

The Collaborative Care Program is developed for patients with severe borderline or NOS personality disorders. When structured psychotherapy for these patients is not sufficient, feasible or available, patients most commonly receive Community Mental Health Care (CMHC). This CMHC is, however, not standardized and relatively unstructured [1,2]. Professional guidelines demonstrate a lack of evidence based recommendations with regard to this sub population of patients with a severe personality disorder. To meet these deficiencies we developed a Collaborative Care Program.

Within Collaborative Care programs nurses have a central coordinating role in optimizing the continuity and coordination of care. They function as a collaborative care manager. Currently, in CMHC nurses typically are the main care providers and they have a central share in the execution of the treatment plan. Research indicates, however, that nurses are not adequately prepared to meet this responsibility [3-6]. Collaborative Care (CC) therefore offers a structured method to support nurses in providing care for patients with severe borderline or NOS personality disorder in community mental health care.

In our previous contribution we described the CC-program briefly and elaborated the research methods for an exploratory study to examine the process of application and the preliminary effects of the intervention program. In this additional file we exemplify the principles and content of this Collaborative Care Program (CCP).

**Collaborative Care Program**

Collaborative Care for patients with severe personality disorders consists of five integrated components:

1. Adequate organisation and coordination of care, with optimal collaboration between the main partners: the patient, his/her informal carers, a psychiatrist and a psychiatric nurse.
2. Reduction of destructive behaviours, (i.e. suicidal behaviours; self-harm; aggression; addiction behaviours) by applying a structured method of early recognition and early intervention;
3. Reduction of daily life problems by the application of Problem Solving Treatment (PST);
4. Realization of a more positive life orientation, by utilizing elements of Solution Focused Treatment;
5. Provision of psycho-education.

The first component refers to the preparation stage of the program, while the components 2, 3, and 4 comprise the treatment stage. An evaluation of process and outcomes of the intervention program takes place every three months based on the goals as described in the treatment plan. Psycho-education is integrated through all stages of the program. The different components will be elucidated in more detail below.

Additionally, during the provision of the CCP, supervision, consultation and coaching for the nurses are provided for permanent education on attitude and skills.

**A collaborative approach**

Within Collaborative Care we prefer an approach in which we perceive the patient as the one who shapes his own life, and hence his own treatment. As care providers we (even literally) take a seat next to the patient when discussing with the patient the priorities and content of treatment, and the process to be followed to accomplish the set treatment goals [7]. The professionals have knowledge and skills available, but it is up to the patient to decide about the kind of the professional support he needs to work on his own goals. Nurses offer, in the role of care manager, all support to the patient to make the best possible decisions. The principle is that no interventions will be executed as long as patient and care providers do not reach consensus about the content and process of treatment. An exception will be made when an acute and inevitable danger for the patient or his/her surrounding is present, as a result of which there is a need to act in line with the Dutch law (Special Admissions Act for Psychiatric Hospitals (BOPZ).

Jobes [7] describes the collaboration as a *therapeutic road trip* in which the patient is the driver and the care provider the navigator. Driver and navigator travel together, but it is the driver who has a decisive vote in where to go and how to get there. The navigator stays right beside the patient with his/her expertise and experience to facilitate the ‘trip’ for a period of time patient and care provider both commit to and to a destination they both agree on. A successful trip requires safety agreements; the trip will fail, if the patient insists on leaving the door ajar so that he/she can jump out of the car if the road gets bumpy. Patients have to stay in the car completely with seat belts on and doors closed and locked. This way of travelling also implies that when the destination is not clear (read: no opinion or consensus about goals of treatment), the departure should be delayed until consensus is reached about the destination. In our program we use this metaphor to illustrate the collaboration between patients and care providers.

This leading principle will undoubtedly be challenged by daily practice. The dynamics of the collaboration occasionally will bring along struggles and conflicts; patients sometimes will not be able or willing to take control over their lives; care providers occasionally feel – more than necessary and possible – responsible for the patient’s life; severe crises can not always be prevented. Nevertheless, the basic attitude still remains: principal acceptance of the patient as a person, hence no judging or condemnation, transparency about and acceptance of restraints of both parties, and joining in the needs or possibilities of the patient.

In the manual, guidelines are elaborated on how to perform this collaborative approach.

**Stage 1: Preparation**

***Organization, collaboration and contracting***

The first stage consists of several steps as a preparation for future treatment and care:

1. Introduction of the principles of Collaborative Care to the patient and informal carers (if appropriate and available) and the forming of a Collaborative Care Team;
2. Evaluation of treatment history, current treatment and collaboration;
3. Systematic assessment of needs;
4. Crisis management;
5. Formulation of a treatment plan.

*a. Introduction of the principles of Collaborative Care and the Collaborative Care Team*

CC is developed for patients who usually have a long history with treatment and with care providers. The successes of these previous efforts and contacts are divergent. Within CC active involvement of the patient is needed to reach the objectives of improved self management and shared decision making. This active involvement, however, is not always obvious. On the one hand patients occasionally are disappointed and suspicious towards (intentions of) care providers due to preceding experiences. Care providers probably have to convince and motivate patients again to become active partners in their own care. It is therefore important to explain to the patient what will be different (and hopefully better) with regard to the collaboration as well as the actual treatment. Nurses, on the other hand, at times have difficulties to facilitate and enhance user involvement and self management skills of patients. Some of them find it hard to lean backwards and not to take responsibility to plan the patient’s goals or solve a patient’s problems. Others may underestimate the needs and disabilities of their patients and perceive them as able but unwilling to change [8-10]. To keep the balance between playing a waiting game on the one hand, and being overly supportive on the other hand is considered to be difficult with regard to these patients [8]. The previously mentioned guidelines with regard to the collaborative approach and the supervision / coaching support nurses to optimize this balance.

To optimize the continuity and coordination of care, a *Collaborative Care Team* (CCT) will be brought together. The CCT consists of the patient, his/her carer, the nurse and the psychiatrist and/or psychologist. Informal carers have a prominent role in the CCT, because we assume that their involvement may benefit treatment. Concurrently, however, it is known that the social or family systems surrounding patients with severe personality disorders are frequently weakened. Over time relationships have suffered from many social and interpersonal problems. In many cases the disrupted relationships within the social system contribute to the psychopathology of the patient and to a low quality of life. In such situations adequate social support for informal carers helps them to persist supporting the patient and to understand and regulate the interactions within the social system. Simultaneously, attention will be paid to diminish the burden of informal carers and provide psycho-education to them.

Next to the members of the CCT, *Collaborative Care partners* are involved. These are all other professionals who are involved in the treatment of the patient, e.g. a general practitioner, a care provider from a specialized addiction service, or a home care nurse. The nurse is responsible to inform and involve all those partners, whenever necessary. Discontinuity of care increases the risk for drop-out and a negative course of the psychiatric illness (with possible suicide as the extreme negative outcome). Transitions between or within health care services, discharge from an inpatient setting, and change of care providers all contribute to these risks. Collaborative Care demands pro-active collaboration between all partners to minimize the risks of this discontinuity.

*b. Evaluation of treatment history, current treatment and collaboration*

Most of the patients have lived a turbulent life due to the occurrence of several (severe) life events. They usually have a long history with health care providers. Life-events and previous experiences with health care providers all affect current relationships and treatment. To learn from previous experiences, an inventory will be made of life events and of all former treatments, based on the medical record. This inventory will be discussed with the patient and with the other members of the CC-Team to identify effective coping strategies with life events, effective elements in treatment, and relationships. Patients are invited to bring along expectations with regard to care providers and treatment and to speak aloud about disappointing (sometimes even traumatic) experiences, e.g. seclusion episodes at closed wards, which still may hamper the relationships with care providers. Informal carers are invited to share their view upon past life events and expectations with regard to collaboration and treatment. The objective is to sustain that what is effective and to give up ineffective approaches. Mutual expectations and responsibilities are made explicit between patients and care providers, in order to promote a strong relationship as one of the main principles of CC. This relationship will be evaluated systematically. During treatment the Session Rating Scale (SRS) and the Outcome Rating Scale (ORS) will be filled in to tune in to expectations and outcomes of each session [11,12].

*c. Systematic assessment of needs*

Perceived needs of patients will be systematically examined by means of the Camberwell Assessment of Need (CAN) [13]. The CAN makes an inventory of 22 human needs, clustered in four categories: psychological, physical, environmental and social. Research indicates that care provider and patient views about these human needs may differ, and thus that their views on needs should be recorded independently [14-16]. In the CCP, the ratings of needs will be completed by care providers and the patient (and their carer) separately. The different perspectives on met and unmet needs will be discussed in the Collaborative Care Team and jointly priorities in treatment will be set. The fulfilment of unmet needs can be aimed at the short or long term, dependent on the urgency and solvability. Long-term goals usually concern future wishes, e.g. building a stable social network or increasing employment opportunities. Goals for the future may inspire and motivate patients to solve or persevere the difficulties in daily life.

Unmet needs, goals and related activities are recorded in the treatment plan, which will be evaluated every three months.

*d. Crisis management*

Life of most patients with personality disorders is dominated by repeated crises. Consequently, attention will be paid to preventing and coping with crises. The use of a crisis card fits the philosophy of collaborative care because it communicates that patients are (at least partly) able to cope with crises themselves. If not, a backup of professional care is always available 24/7 [7].

A crisis card consists of five activities for patients to carry out when a crisis occurs. The crisis card ideally is completed by the patient and includes concrete activities to reduce stress or despair. Prior to the drafting of the crisis card, the occurrence and nature of individual crises will be discussed with the patient and their informal carers: when do crises occur, how do crises develop over time, how can they best be solved, what does the patient expect from others (informal carers and professionals) during a crisis? In anticipation of severe crisis, the possibility and desirability of an admission will be discussed. The duration, expectations, and goals of a possible admission will be recorded.

*e. Formulation of a treatment plan*

Finally, at the end of the contracting stage a treatment plan is formulated, to which all partners involved commit and which meets the individual needs of the patient. Contracting is perceived as the obvious means for patients with personality disorders to exemplify mutual expectations and responsibilities [7,17]. Contracting includes shared decision making about the collaboration, form as well as the content of treatment. Conflicting views are discussed and neutralised if possible, in order to create and maintain a predictable supportive structure for treatment, which is watched over, regardless of frequent challenges to release this structure.

**Stage 2: Execution**

***Destructive behaviour***

Patients with borderline or NOS personality disorders often show various or multiple problem behaviours. These problem behaviours can be expressed in several ways: suicidal, self harm, addictive or aggressive behaviour. A method of early recognition and early intervention will be used to gain insight in the process of emergent problem behaviour and to enhance coping with this destructive behaviour. Early recognition plans are drawn up concerning different kinds of destructive behaviour [7,18-20].

The central aim is the early recognition of triggers and signs (thoughts, feelings or behaviours) of the destructive behaviour of the patient. Experience shows that in general destructive behaviour does not originate out of nowhere (and thus unexpectedly), but that a gradual process of rising stress or despair precedes it (figure 1). Specific risks or situations (triggers) can be identified in advance, as a result of which the behaviour occurs. Individual signs of rising stress or despair can be explored, e.g. bad sleep, thoughts of failure or self-hate, withdrawal from social contacts by not answering the phone, or cancelling appointments. Occasionally, hours, days or even weeks pass between the first signs of stress and the moment of escalation when the destructive behaviour arises severely. Especially this period offers opportunities for preventive interventions.

Nurses support the patient to gain insight in his/her individual process of increasing stress and despair leading to specific destructive behaviour. The first step is to explore the different destructive behaviours of each patient. Self report questionnaires, such as the Beck Scale for Suicide Ideation [21], the Self Harm Questionnaire [22], the CAGE-AID [23] and questions regarding aggressive behaviour are used for this exploration and for raising the awareness of destructive behaviour. Secondly, the triggers and early signs will be identified and recorded in the relapse prevention plan. Subsequently, actions will be chosen to react adequately at an imminent crisis. Actions to reduce stress or despair will ideally take place as early as possible. The better the patient recognizes his/her specific triggers and early signs, the better the patient can act proactively (preferably in stage S1: see figure 1). Part of the early recognition plan is the crisis card for those moments when the stress rises too high and acute acting is needed to prevent damage and to promote control (stage S2).

The incidence of specific destructive behaviour will be assessed repeatedly by means of the self-report questionnaires. This makes it possible to discuss despair and corresponding destructive behaviours openly and systematically, and to periodically evaluate and update the relapse prevention plan based on new insights. This method of early recognition and intervention can be individualised according to the level of cognitive and social functioning of the individual patient. Informal carers are invited to contribute to the development of this relapse prevention plan.

***Problem Solving***

In the preparation stage an inventory is made of individual needs with the Camberwell Assessment of Need (CAN). Subsequently, in the treatment plan goals are formulated based on the unmet needs. Many of these needs tend to correspond with some of the daily problems the patient perceives, like finding significant day activities, coping with debts, housing problems, interpersonal problems et cetera. The amount of problems is often so overwhelming that patients are paralysed by it and may loose their sense of control. To enhance the self-management skills of the patient in solving these problems, Problem Solving Treatment (PST) will be provided. Learning and applying problem solving skills regarding daily problems enhances patients’ feelings of mastery. Mastery reflects the extent to which individuals perceive themselves to be in control of forces that significantly impact their lives. PST has proven to be effective in various studies and is part of different treatments for personality disorders [17,24-26]. PST diminishes mental disorders and decreases indirect costs. It is an essential element of Collaborative Care programs [27-29].

***Life orientation***

As a counterbalance to the prominent attention to problems, the focus is also aimed at a more positive orientation in a person’s life. To encounter and expand positive experiences might be stimulating for a renewed and more positive life orientation.

A lack of positivity seems to be related to cluster B personality disorders [30]. This can be explained by: (1) a lack of rewarding and enjoyable experiences, (2) a cognitive impairment of representations of a positive future, (3) an inability to derive pleasure from what are normally enjoyable events [30]. A more positive life orientation, with increased optimism and reduced hopelessness may protect against suicidal behaviour [30-33]. To achieve this we introduce some exercises to expand positive experiences and to visualize a more stimulating future. We focus on and stimulate the unique qualities and strengths of people. The exercises are based on the principles of solution focused treatment [34-36].

***Psycho education***

By means of psycho education, the patient (and his informal carer) is provided with knowledge about his or her psychiatric disorder and psychological condition, the causes and consequences, effective ways of coping, and the treatment possibilities including their expected effects. Psycho education is an integral element of Collaborative Care. Throughout all stages of the program nurses examine whether the patients and their informal carers have sufficient knowledge of the illness and its consequences. Patients’ personal experiences are related to corresponding symptoms of the illness. Patients are taught how to cope effectively with the consequences of their illness in daily life. Patients and their carers also will be prepared to the enduring character of the illness and to expected relapses. The main psycho educational information is written out in the workbook for patients. Supplementary resources (books or websites) are listed.

**Stage 3: Evaluation**

The treatment plan will be evaluated in the Collaborative Care Team every three months. This enables the patient to monitor his progress and - if necessary - change treatment decisions and reformulate goals. During the evaluation, the collaboration between the patient and the members of the CC-Team will also be evaluated. The perspectives and experiences of all CCT members during the last period will be clarified. Based on this evaluation the treatment plan will be adjusted.

**Discussion**

With this Collaborative Care Program we aim to support patients, their informal carers and care providers in the organisation and provision of effective treatment. We assume that the easily accessible structured CC Program will have an additional value compared to CAU. When compared to some psychotherapies, like Dialectical Behavioural Therapy [17], mentalization-based treatment [37], schema-focused and transference-focused treatment [38], the CCP is more easily accessible and less intensive. The primary objective of these psychotherapies differs from that of CC. Psychotherapy is aimed at sustainable changes in personality, psychopathology and recovery, while CC is aimed at increased self-management of chronic illnesses and improved quality of life. Primary choice of treatment for most patients with personality disorders, in accordance with international multidisciplinary guidelines, should be psychotherapy. This CCP is developed for the subpopulation of patients who, for different reasons, do not have access to this psychotherapy. For this subpopulation the CCP might be an alternative for the care as usual within community mental health care.

Within the CCP the pro-active involvement of all collaborative partners, including informal carers, will probably contribute to the effectiveness and continuity of care provided. For nurses this implies a more pro-active anticipating strategy, because they are the ones who inform and involve different partners. The investments in time and effort during the preparation stage especially will probably be earned back over time. Crisis management, contracting and shared goals to which all partners commit prevent misunderstandings, needless treatment and needless admissions at both emergency and inpatient settings.

Furthermore, the collaborative approach supports patient and nurse to sustain an effective relationship, which will be systematically evaluated. The collaborative approach challenges nurses to lean back and not take too much responsibility for the patient’s life. At the same time, this approach tempts patients to be an active partner in their care and to take responsibility for their choices, goals and activities within treatment. The CCP has a goal-oriented structure, which means that in each session these goals need to be made explicit. An endless offer without commitment, which at times characterise community mental health care, makes patients unnecessarily dependent on mental health care and decreases their autonomy. In the context of the CCP nurses are trained to increase their alertness of this goal-oriented structure over time and in each session. During the training of the CCP nurses will be taught how to accomplish their role as care manager with regard to the specific subpopulation of patients. The supervision, coaching and consultation offer additional support to maintain and expand their skills.

To conclude, with this CC Program nurses are offered knowledge, skills and structure to be better equipped to provide care for these patients. As we have seen, nurses are the main care providers within CMCH teams for this sub population of patients, which nurses perceive occasionally as ‘difficult’ patients. Collaborative Care underlines the coordinating role of nurses within the multidisciplinary collaboration. It provides a framework for care providers in which collaboration, coordination and continuity of care and treatment are integrated.

Reference List

1. Koekkoek B, Meijel van B, Schene A, Hutschemaekers G: **Community psychiatric nursing in the Netherlands: a survey of a thriving but threatened profession.** *J Psychiatr Ment Health Nurs* 2009, **16:** 822-828.

2. Koekkoek B, Meijel van B, Hutschemaekers G: **Community mental health care for people with severe personality disorder: a narrative review.** *The Psychiatrist* 2010, **34:** 24-30.

3. Deans C, Meocevic E: **Attitudes of registered psychiatric nurses towards patients diagnosed with borderline personality disorder.** *Contemp Nurse* 2006, **21:** 43-49.

4. Fraser K, Gallop R: **Nurses' confirming/disconfirming responses to patients diagnosed with borderline personality disorder.** *Arch Psychiatr Nurs* 1993, **7:** 336-341.

5. Markham D, Trower P: **The effects of the psychiatric label 'borderline personality disorder' on nursing staff's perceptions and causal attributions for challenging behaviours.** *Br J Clin Psychol* 2003, **42:** 243-256.

6. Newton-Howes G, Weaver T, Tyrer P: **Attitudes of staff towards patients with personality disorder in community mental health teams.** *Aust N Z J Psychiatry* 2008, **42:** 572-577.

7. Jobes DA: *Managing Suicidal Risk. A Collaborative Approach.* New York: The Guilford Press; 2006.

8. Koekkoek B, Meijel van B, Tiemens B, Schene A, Hutschemaekers G: **What makes community psychiatric nurses label non-psychotic chronic patients as 'difficult': patient, professional, treatment and social variables.** *Soc Psychiatry Psychiatr Epidemiol* 2010.

9. Koekkoek B, Meijel van B, Schene A, Hutschemaekers G: **Problems in psychiatric care of 'difficult patients': a Delphi-study.** *Epidemiol Psichiatr Soc* 2009, **18:** 323-330.

10. Koekkoek B, Meijel van B, Schene A, Hutschemaekers G: **Clinical problems in community mental health care for patients with severe borderline personality disorder.** *Community Ment Health J* 2009, **45:** 508-516.

11. Miller SD, Duncan BL, Brown J, Sparks JA, Claud DA: **The Outcome Rating Scale: A preliminary study of the reliability, validity and feasibility of a brief visual analog measure.** *Journal of brief Therapy* 2003, **2:** 91-100.

12. Duncan B.L., Miller SD, Sparks JA, Claud DA, Reynolds LR, Brown J, Johnson LD: **The Session Rating Scale: Preliminary psychometric properties of a "working" alliance measure.** *Journal of brief Therapy* 2003, **3:** 3-12.

13. Phelan M, Slade M, Thornicroft G, Dunn G, Holloway F, Wykes T, Strathdee G, Loftus L, McCrone P, Hayward P: **The Camberwell Assessment of Need: the validity and reliability of an instrument to assess the needs of people with severe mental illness.** *Br J Psychiatry* 1995, **167:** 589-595.

14. Lasalvia A, Bonetto C, Malchiodi F, Salvi G, Parabiaghi A, Tansella M, Ruggeri M: **Listening to patients' needs to improve their subjective quality of life.** *Psychol Med* 2005, **35:** 1655-1665.

15. Lasalvia A, Bonetto C, Tansella M, Stefani B, Ruggeri M: **Does staff-patient agreement on needs for care predict a better mental health outcome? A 4-year follow-up in a community service.** *Psychol Med* 2008, **38:** 123-133.

16. Slade M: **Needs assessment. Involvement of staff and users will help to meet needs.** *Br J Psychiatry* 1994, **165:** 293-296.

17. Linehan MM: *Cognitive-Behavioral Treatment of Borderline Personality Disorder*. New York: The Guilford Press; 1993.

18. Bosman M., Meijel van B: *Begeleiding van patiënten die zelfverwonden: een verpleegkundig interventiepakket. [Treatment of patients who self harm: a nursing intervention tool]*. Maarssen: Elsevier Gezondheidszorg; 2009.

19. Fluttert F, Meijel van B, Webster C, Nijman H, Bartels A, Grypdonck M: **Risk management by early recognition of warning signs in patients in forensic psychiatric care.** *Arch Psychiatr Nurs* 2008, **22:** 208-216.

20. Meijel van B, Gaag van der M, Kahn RS, Grypdonck MH: **Relapse prevention in patients with schizophrenia: the application of an intervention protocol in nursing practice.** *Arch Psychiatr Nurs* 2003, **17:** 165-172.

21. Beck AT, Steer RA, Ranieri WF: **Scale for Suicide Ideation: psychometric properties of a self-report version.** *J Clin Psychol* 1988, **44:** 499-505.

22. Claes L, Vandereijken W, Vertommen H. Zelfverwondingsvragenlijst [Self harm questionnaire]. 2004.

23. Brown RL, Rounds LA: **Conjoint screening questionnaires for alcohol and other drug abuse: criterion validity in a primary care practice.** *Wis Med J* 1995, **94:** 135-140.

24. Mynors-Wallis L, Davies I, Gray A, Barbour F, Gath D: **A randomised controlled trial and cost analysis of problem-solving treatment for emotional disorders given by community nurses in primary care.** *Br J Psychiatry* 1997, **170:** 113-119.

25. Black DW, Blum N, Eichinger L, McCormick B, Allen J, Sieleni B: **STEPPS: Systems Training for Emotional Predictability and Problem Solving in women offenders with borderline personality disorder in prison--a pilot study.** *CNS Spectr* 2008, **13:** 881-886.

26. Blum N, St JD, Pfohl B, Stuart S, McCormick B, Allen J, Arndt S, Black DW: **Systems Training for Emotional Predictability and Problem Solving (STEPPS) for outpatients with borderline personality disorder: a randomized controlled trial and 1-year follow-up.** *Am J Psychiatry* 2008, **165:** 468-478.

27. IJff MA, Huijbregts KM, Marwijk van HW, Beekman AT, Hakkaart-Roijen van L, Rutten FF, Unutzer J, Feltz-Cornelis van der CM: **Cost-effectiveness of collaborative care including PST and an antidepressant treatment algorithm for the treatment of major depressive disorder in primary care; a randomised clinical trial.** *BMC Health Serv Res* 2007, **7:** 34.

28. Vlasveld MC, Anema JR, Beekman AT, Mechelen van W, Hoedeman R, Marwijk van HW, Rutten FF, Hakkaart-Roijen van L, Feltz-Cornelis van der CM: **Multidisciplinary collaborative care for depressive disorder in the occupational health setting: design of a randomised controlled trial and cost-effectiveness study.** *BMC Health Serv Res* 2008, **8:** 99.

29. Bodenheimer T, Lorig K, Holman H, Grumbach K: **Patient self-management of chronic disease in primary care.** *JAMA* 2002, **288:** 2469-2475.

30. MacLeod AK, Tata P, Tyrer P, Schmidt U, Davidson K, Thompson S: **Personality disorder and future-directed thinking in parasuicide.** *J Pers Disord* 2004, **18:** 459-466.

31. MacLeod AK, Tata P, Tyrer P, Schmidt U, Davidson K, Thompson S: **Hopelessness and positive and negative future thinking in parasuicide.** *Br J Clin Psychol* 2005, **44:** 495-504.

32. Soloff PH, Lynch KG, Kelly TM, Malone KM, Mann JJ: **Characteristics of suicide attempts of patients with major depressive episode and borderline personality disorder: a comparative study.** *Am J Psychiatry* 2000, **157:** 601-608.

33. Malone KM, Oquendo MA, Haas GL, Ellis SP, Li S, Mann JJ: **Protective factors against suicidal acts in major depression: reasons for living.** *Am J Psychiatry* 2000, **157:** 1084-1088.

34. de Shazer S, Berg IK, Lipchik E, Nunnally E, Molnar A, Gingerich W, Weiner-Davis M: **Brief therapy: focused solution development.** *Fam Process* 1986, **25:** 207-221.

35. McAllister M, Zimmer-Gembeck M, Moyle W, Billett S: **Working effectively with clients who self-injure using a solution focused approach.** *Int Emerg Nurs* 2008, **16:** 272-279.

36. Wand T: **Mental health nursing from a solution focused perspective.** *Int J Ment Health Nurs* 2010, **19:** 210-219.

37. Allen JG, Fonagy P, Bateman AW: *Mentalizing in Clinical Practice.* Washington D.C.: American Psychiatric Publishing; 2008.

38. Giesen-Bloo J, Dyck van R, Spinhoven P, Tilburg van W, Dirksen C, Asselt van T, Kremers I, Nadort M, Arntz A: **Outpatient psychotherapy for borderline personality disorder: randomized trial of schema-focused therapy vs. transference-focused psychotherapy.** *Arch Gen Psychiatry* 2006, **63:** 649-658.

**Supplementary figures**

Figure 1: the process of rising stress/despair.

Stress/despair

time

S0

S2

S3

Problem

behaviour

threshold

S1

10

0
